# Cardiometabolic risk factor levels in Norwegian children compared to international reference values: The ASK study

**DOI:** 10.1371/journal.pone.0220239

**Published:** 2019-08-19

**Authors:** Mette Stavnsbo, Turid Skrede, Eivind Aadland, Katrine N. Aadland, Mai Chinapaw, Sigmund A. Anderssen, Lars B. Andersen, Geir K. Resaland

**Affiliations:** 1 Department of Sport, Food and Natural Sciences, Western Norway University of Applied Sciences, Sogndal, Norway; 2 Department of Sports Medicine, Norwegian School of Sport Sciences, Oslo, Norway; 3 Department of Public and Occupational Health, Amsterdam University Medical Centers, Vrije Universiteit Amsterdam, Amsterdam Public Health research institute, Amsterdam, The Netherlands; 4 Center for Physically Active Learning, Faculty of Education, Arts and Sports, Western Norway University of Applied Sciences, Sogndal, Norway; University of Western Australia, AUSTRALIA

## Abstract

**Objective:**

To investigate cardiometabolic risk factor levels in a group of Norwegian 10-year-old children compared to international values and examine the association between cardiorespiratory fitness (CRF) and the reference-standardized clustered risk score.

**Methods:**

913 children (49% girls) were included from the Active Smarter Kids (ASK) study. Body mass index (BMI), waist circumference (WC), systolic blood pressure (SBP), diastolic blood pressure (DBP), low-density lipoprotein cholesterol (LDL-C), high-density lipoprotein cholesterol (HDL-C), total cholesterol (TC) to HDL-C ratio, triglyceride (TG), glucose, insulin, homeostatic model assessment (HOMA) score and CRF, were standardized according to international age-and sex-specific reference values.

**Results:**

The Norwegian children had significantly more favorable WC, DBP, glucose, HDL-C and CRF levels compared to the international reference values, but similar or less favorable levels of other cardiometabolic risk factors. CRF was the variable that differed the most from the international values (mean (95% CI) 1.20 (1.16 to 1.24) SD). The clustered risk score (excluding CRF) was higher in the Norwegian children, but decreased to below international levels when including CRF (mean (95% CI) - 0.08 (- 0.12 to –0.05) SD). CRF had a significant inverse association with the clustered risk score (excluding CRF) (β - 0.37 SD, 95% CI –0.43 to –0.31).

**Conclusions:**

Norwegian children have substantially higher CRF levels than international standards, and including CRF in clustered risk scores reduces overall risk in Norwegian children below that of international levels. CRF is associated with improved cardiometabolic health in children.

## Introduction

The clustering of cardiometabolic risk factors (hypertension, dyslipidemia, adiposity, and glucose intolerance) has its origin in childhood [1] and can track into adulthood [2].

These risk factors constitute the metabolic syndrome (MetS), but the use of different measures and ensembles of risk factors and thresholds [3–5] hampers comparison between studies. Further, dichotomization of risk factors to define cardiometabolic risk have several limitations, especially when carried out in children [6]. The dichotomization of biological traits ignores the continuous nature of risk and decreases the available information and thus power in statistical analysis. Moreover, since cardiovascular diseases are usually not manifested in children, the thresholds for classifying children as “*healthy*” or “*at risk*” [1, 7–9] are adapted from definitions in adults and introduce an arbitrary insincerity.

A large international reference material for cardiometabolic risk factor values in children and adolescents was recently published to deal with these issues [10]. The reference values can be used to standardize single cardiometabolic risk factors, allowing to compare otherwise population specific continuous clustered risk scores directly to the reference material itself and to other studies adapting the same approach. Thus, the study by Stavnsbo et al. [10] facilitate international comparisons of prevalence and trends in pediatric cardiometabolic risk.

Cardiorespiratory fitness (CRF) is a strong predictor of cardiometabolic disease and all-cause mortality in adults [11, 12]. In children, the evidence is less clear but CRF has been inversely associated with clustered cardiometabolic risk factors in an accumulating number of studies [13–17]. The primary aim of this paper was to investigate cardiometabolic risk factor levels in a group of Norwegian 10-year-old school-children as compared to international reference values. A secondary aim was to examine the association between CRF and the reference-standardized clustered risk score.

## Materials and methods

### Design and population

The present study is a cross-sectional analysis of baseline data from the Active Smarter Kids (ASK) study, a seven month cluster-randomized controlled trial conducted in the school year of 2014–2015 in Western Norway [18]. In total, 1129 5^th^ graders (94% of those invited) from 57 schools participated in the ASK study. We included 913 children (49% girls vs. 51% boys) age 10.2 (± 0.3) that had valid data in all cardiometabolic risk factors of interest. The ASK study design, sampling procedures and methodology is described in details elsewhere [18], thus, only a brief description of the relevant procedures are provided herein.

### Blood sampling

An intravenous blood sample was collected from the children’s antecubital vein after an overnight fast. Serum were obtained following a standardized protocol. An ISO-certificated laboratory analyzed the serum samples for traditional risk factors related to cardiometabolic diseases; insulin, glucose, triglycerides (TG), high density lipoprotein cholesterol (HDL-C), low density lipoprotein cholesterol (LDL-C), and total cholesterol (TC). A TC to HDL-C (TC:HDL-C) ratio was calculated to represent dyslipidemia. Insulin resistance were defined by the homeostatic model assessment (HOMA)-score = [insulin (pmol/L) * glucose (mmol/L)]/135 [19].

### Resting blood pressure

Systolic (SBP) and diastolic (DBP) blood pressure were measured by the Omron HEM-907 automated BP monitor (Omron Healthcare, Inc, Vernon Hills, IL, US). The device is validated according to the AAMI validation protocol [20] and to the validation criteria of the international protocol for BP measuring devices [21]. The children were measured in a quiet room after resting for ten minutes in a sitting position. Four measurements were taken with one-minute pauses in-between and the mean of the last three measurements was used for analyses. If the difference between measurements was >5 mmHg, we obtained one extra measurement, in which case the mean of the last four was calculated and used for analyses.

### Anthropometry and sexual maturity

#### BMI

Body mass was measured to the nearest 0.1 kg using an electronic scale (Seca 899, SECA GmbH, Hamburg, Germany) with children wearing light clothing or underwear (preferred) depending on the acceptance of the child. A portable Seca 217 (SECA GmbH, Hamburg, Germany) was used to measure stature to the nearest 0.1 cm with the barefooted child facing forward. Body mass index (BMI) (kg*m^−2^) was calculated as weight (kg) divided by the height squared (m^2^).

#### Waist circumference

Waist circumference (WC) was measured using an ergonomic measuring tape (Seca 201, SECA GmbH, Hamburg, Germany). Two measures were taken between the lowest rib and the iliac crest to the nearest 0.5 cm with the child’s abdomen relaxed at the end of a gentle expiration. If the difference between measurements was greater than one cm, we obtained a new measurement until two results were ≤ 1 cm apart. The mean of the two closest measurements was used for analyses.

#### Maturity

Pubertal stage was self-assessed by the child using the Tanner scale in color pictures as proposed by Carel and Leger [22]. The children were given the standardized series of images with explanatory text in a private room. The children were asked to put a checkmark in the box below the picture that best represented their stage (1 to 5) of development for each component. We used breast and genital development for girls and boys, respectively, as a measure of pubertal stage.

### Demographic characteristics

We obtained self-reported educational level from the children’s parents/legal guardians to assess socio-economic status (SES). Parental education was categorized into three levels using the highest educational level obtained by the mother or father: i) upper or lower secondary school, ii) university < four years, and iii) university ≥ four years.

### Cardiorespiratory fitness

Cardiorespiratory fitness levels were measured using the validated Andersen test [23, 24], an intermittent field-running test [23]. All children were tested indoor on a wooden or rubber hall floor. In groups of 10–20 children, participants ran a 20 meter distance between one end-line to another for 15 seconds and stood still for another 15 seconds. Children touched the floor with one hand behind the line each time before they turned around. The test lasted for 10 minutes, and the total distance (meters) covered was registered as the test result. The Andersen test performance was converted into VO_2peak_ by the following equation; girls VO_2peak_ = 32.5793 + (0.0309 × distance (m))–(0.2351 × body mass (kg)), boys VO_2peak_ = 27.1689 + (0.0397 × distance (m))–(0.1698 × body mass (kg)) [25].

### Ethics

Our procedures and methods conform to ethical guidelines defined by the World Medical Association's Declaration of Helsinki and subsequent revisions [26]. The study protocol was approved by the Regional Committee for Medical Research Ethics (Reference Number: 2012/2304). Written informed consent was obtained from children’s parents or legal guardians prior to commencement to the study.

### Statistics

Before analyses, all values exceeding five standard deviations from the mean were excluded from the data material. Skewed variables were logarithmically transformed by the natural logarithm (ln); BMI, WC, TG, TC:HDL-ratio, insulin and HOMA. A linear mixed model and a generalized estimating equation model including school as a random effect were used to examine differences between sexes for the continuous and categorical variables, respectively. The characteristics are presented as means and standard deviations (SD), median and interquartile range (IQR), or numbers and percentage (%). To enable comparison of single and clustered cardiometabolic risk factor values between Norwegian children and the international reference values, we standardized the following risk factors according to the age- and sex-specific reference values suggested by Stavnsbo et al. [10]; BMI (ln), WC (ln), SBP, DBP, LDL-C, HDL-C, TC:HDL-ratio (ln), TG (ln), glucose, insulin (ln), HOMA score (ln), and CRF (VO_2peak_). Each single risk variable was standardized by sex using the following equation; reference-standardized variable (z-score) = $\left(x+\bar{x})/\mathrm{SD}(\bar{x}\right)$, where age-predicted reference values were used as the mean ($\bar{x}$), calculated from regression equations for the single cardiometabolic risk factors [10]. A mean clustered reference-standardized risk score was calculated by summing up the z-scores WC, SBP, TC:HDL-ratio, TG, and HOMA score and divide by five [27]. A second mean clustered risk score was calculated including CRF (VO_2peak_ inversed) by summing up the same reference-standardized risk factors as described above and CRF, and divide by six [27].

The association between the Andersen test and the reference-standardized clustered risk score (excl. CRF) was explored using a linear mixed model; CRF as the independent variable, the reference-standardized clustered risk score as the dependent variable, and school as a random effect (to account for the cluster effect). Age, sex, pubertal stage and SES were included as covariates, but only sex and pubertal stage changed the estimates and were therefore included in an adjusted model. In line with previous studies [14, 15], we investigating if sex moderated the association between CRF and clustered cardiometabolic risk factors, testing the statistical additive assumption in linear regression analysis. Thus, sex differences were investigated by including the interaction CRF*sex. To produce interpretable beta coefficients, both CRF and the clustered risk score were standardized before analysis.

All analyses were conducted using IBM SPSS version 23 (IBM SPSS Statistics for Windows, Armonk, NY: IBM Corp., USA). A *p*-value ≤ 0.05 was considered statistically significant in all analyses.

## Results

Table 1 presents descriptive statistics of the study population. The majority of the children were pre-pubertal at baseline (88% girls and 89% boys) and 63–64% of both girls and boys had at least one parent with a professional bachelor degree (<4 years of higher education). There were no sex differences in mean age, height, BMI, WC, SBP, DBP or LDL. Girls had a significantly higher TC:HDL-ratio, TG, insulin, and HOMA score than boys, but lower CRF levels. Overall, girls had a less favorable risk score profile, represented by a higher clustered risk score (standardized by population specific means and SDs) than boys *(p*<0.001) (results not shown).

**Table 1:** Descriptive statistics of the study population by sex.

	Girls (*n =* 446)	Boys (*n =* 467)	
	Mean (±SD)/ median [Q1-Q3]/n (%)	Mean (±SD)/ median [Q1-Q3]/n (%)	*p*-value
Age (yr)	10.2 (0.3)	10.2 (0.3)	0.885
Puberty (tanner) n (%)			0.001
Stage 1	99 (22.2)	169 (36.2)	
Stage 2	292 (65.5)	248 (53.1)	
Stage 3–5	52 (11.7)	48 (10.3)	
Missing	3 (0.6)	2 (0.4)	
Parents`education level n (%)			0.888
≤ Upper secondary school	148 (33.2)	146 (31.3)	
<4 years of university	122 (27.4)	141 (30.2)	
≥4 years of university	159 (35.6)	159 (34.0)	
Missing	17 (3.8)	21 (4.5)	
Weight (kg)	37.1 (8.3)	37.0 (7.9)	0.941
Height (cm)	142.5 (6.8)	143.1 (6.7)	0.111
BMI (kg/m^2^)	17.3 [15.9–19.6]	17.2 [15.8–19.4]	0.379
WC (cm)	59.6 [56.0–65.3]	60.8 [57.3–65.8]	0.061
SBP (mm Hg)	105.3 (8.5)	105.3 (8.2)	0.669
DBP (mm Hg)	58.1 (6.3)	57.4 (6.1)	0.095
LDL-C (mmol/L)	2.52 (0.62)	2.50 (0.67)	0.615
HDL-C (mmol/L)	1.55 (0.35)	1.63 (0.34)	0.001
TC:HDL-ratio	2.82 [2.48–3.37]	2.72 [3.00–3.12]	0.001
TG (mmol/L)	0.73 [0.58–0.96]	0.65 [0.52–0.83]	<0.001
Glucose (mmol/L)	4.94 (0.33)	5.02 (0.32)	<0.001
Insulin (pmol/L)	52.8 [39.0–75.4]	45.4 [32.7–60.8]	<0.001
HOMA score	1.93 [1.37–2.83]	1.67 [1.19–2.29]	<0.001
Andersen test (m)	870.1 (84.7)	922.9 (111.7)	<0.001
Estimated VO_2peak_ (ml/kg/min)	50.49 (3.1)	57.08 (4.7)	<0.001

BMI; body mass index, CRF; cardiorespiratory fitness, DBP; diastolic blood pressure, HDL-C; high-density lipoprotein cholesterol, HOMA; homeostatic model assessment, LDL-C; low-density lipoprotein cholesterol, n; number, SBP; systolic blood pressure, SD; standard deviation, TC; total cholesterol, TG; triglycerides, WC; waist circumference. A *p*-value ≤0.05 was considered statistical significant.

**Fig 1:**
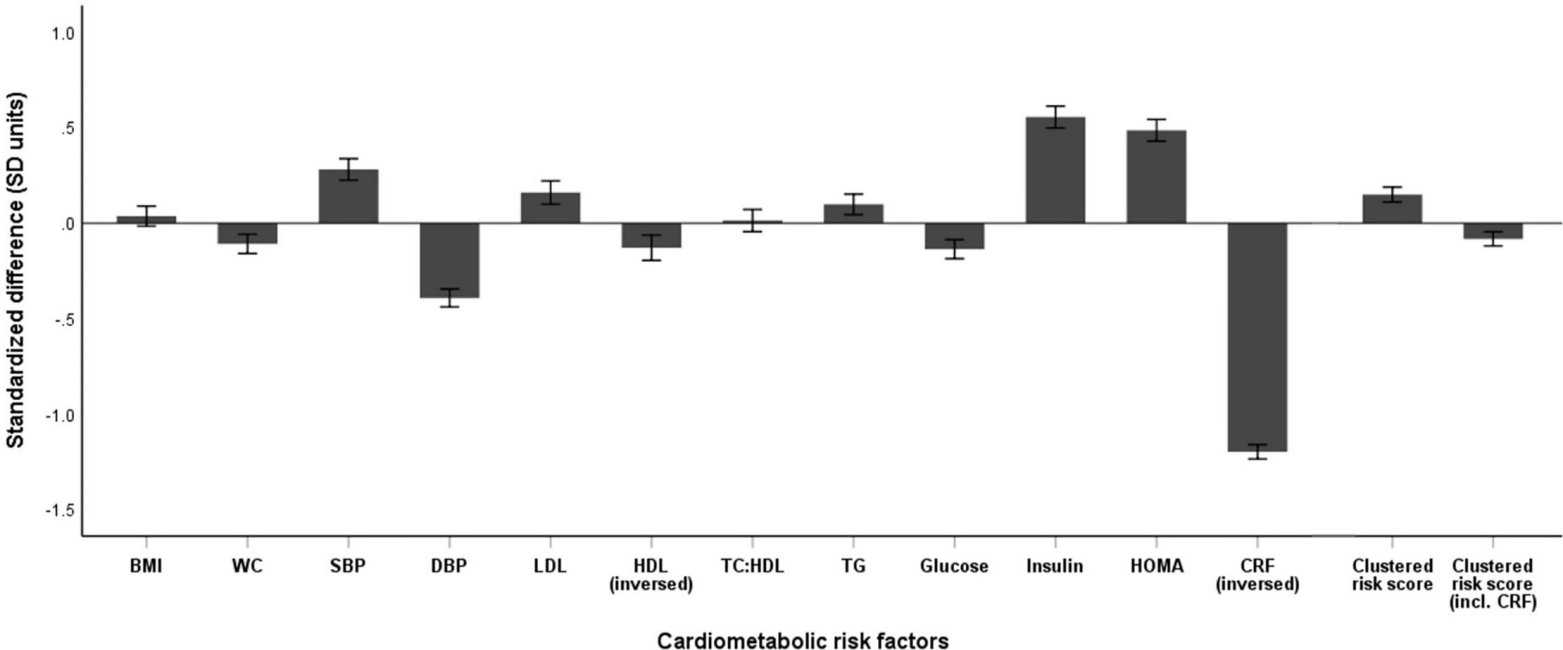
Reference-standardized cardiometabolic risk factors. Mean (95% CI) of the reference-standardized single risk factors and mean clustered risk scores excluding and including cardiorespiratory fitness (CRF) (inversed) in Norwegian children. Reference-standardized variable = $\left(x+\bar{x})/\mathrm{SD}(\bar{x}\right)$, where age-predicted reference value was used as the mean ($\bar{x}$) (10). The cardiometabolic clustered risk scores consisted of the following reference-standardized risk factors; WC, SBP, TG, TC:HDL-ratio, and HOMA score, excluding and including CRF (inversed).

Table 2 and Fig 1 shows the standardized difference in cardiometabolic risk factors and clustered risk scores between Norwegian children and international reference values from children of the same age and sex. Cardiorespiratory fitness differed the most of all standardized variables from the international reference values, showing significantly more favorable levels in the Norwegian children (mean (95% CI) 1.20 (1.16 to 1.24) SD). The Norwegian children also had significantly more favorable WC, DBP, HDL-C, and glucose levels in comparison to the international reference population. Less favorable levels were found for SBP, LDL-C, TG, insulin and HOMA score compared to the international standards. BMI and TC:HDL-ratio was not significantly different from the reference values. The clustered risk score (excluding CRF) was significantly higher in the Norwegian children compared to international values (mean (95% CI) 0.15 (0.11 to 0.19) SD). On the contrary, when including CRF as an additional risk factor, the mean clustered risk score decreased to below international levels (mean (95% CI) - 0.08 (- 0.12 to –0.05) SD).

**Table 2:** Reference-standardized cardiometabolic risk factors^a^ by gender according to Stavnsbo et al. [10].

	Girls	Boys
	Mean (±SD)	Mean (±SD)
BMI	0.03 (0.88)	0.03 (0.87)
WC	- 0.14 (0.89)	- 0.10 (0.81)
SBP	0.36 (0.99)	0.20 (0.88)
DBP	-0.39 (0.81)	-0.43 (0.75)
LDL-C	0.10 (0.94)	0.20 (1.05)
HDL-C	0.10 (1.08)	0.16 (1.06)
TC:HDL-ratio	0.03 (0.99)	- 0.02 (0.89)
TG	0.14 (0.95)	0.04 (0.80)
Glucose	-0.16 (0.84)	-0.13 (0.77)
Insulin	0.58 (1.00)	0.49 (0.81)
HOMA score	0.50 (0.99)	0.43 (0.82)
CRF	1.20 (0.56)	1.23 (0.66)
Clustered risk score ^b^	0.18 (0.67)	0.11 (0.55)
Clustered risk score incl. CRF ^b^	- 0.05 (0.62)	- 0.11 (0.52)

BMI; body mass index, CRF; cardiorespiratory fitness, DBP; diastolic blood pressure, HDL-C; high-density lipoprotein cholesterol, HOMA; homeostatic model assessment, LDL-C; low-density lipoprotein cholesterol, SBP; systolic blood pressure, SD; standard deviation, TC; total cholesterol, TG; triglycerides, WC; waist circumference.

^a^ Reference-standardized variable = $\left(x+\bar{x})/\mathrm{SD}(\bar{x}\right)$, where age-predicted reference values were used as the mean ($\bar{x}$) [10].

^b^ The clustered risk scores was calculated from the following reference-standardized variables; WC, SBP, TC:HDL-ratio, TG, and HOMA score, excluding and including CRF (VO_2peak_ inversed).

**Fig 2:**
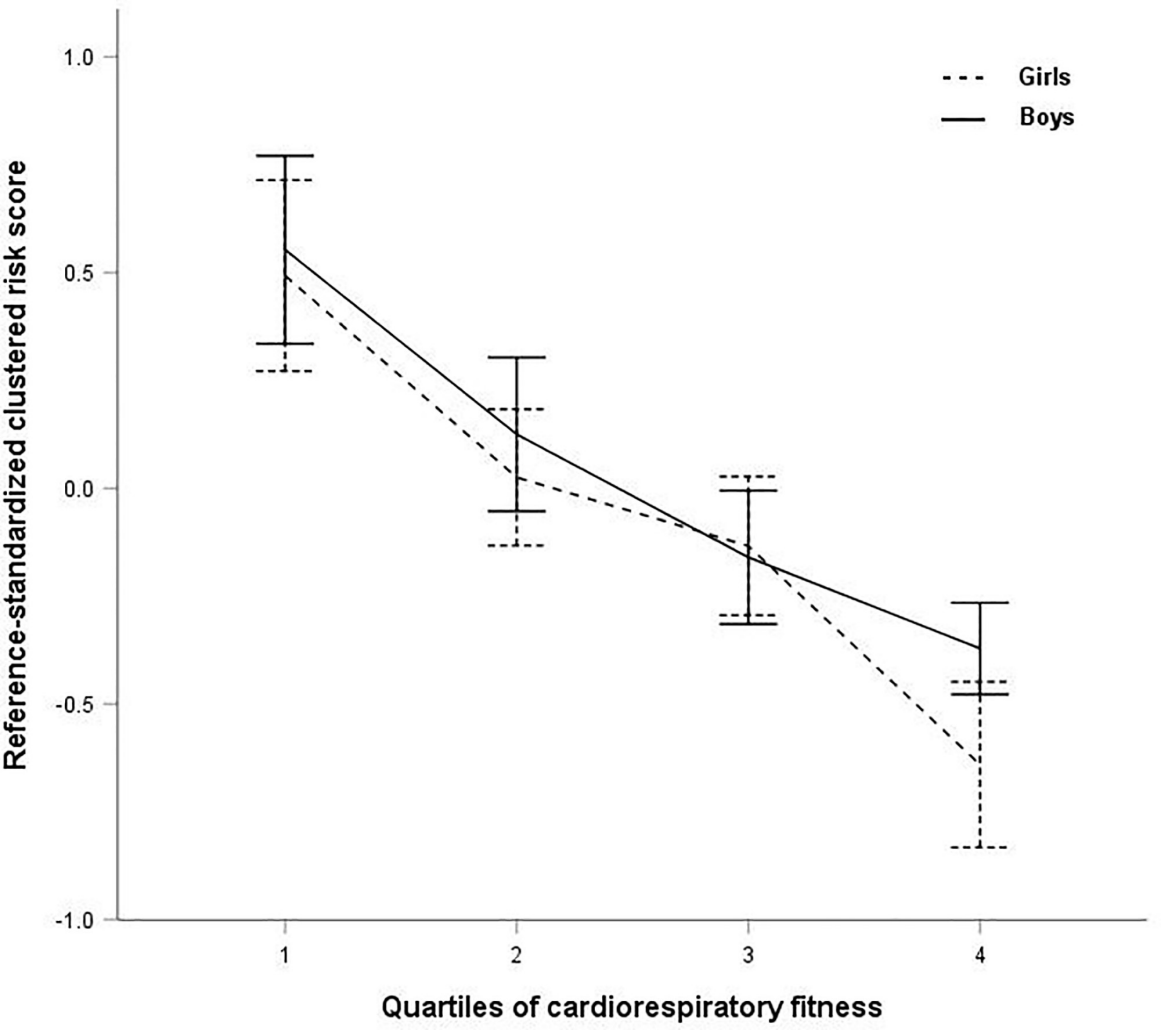
Association between quartiles of cardiorespiratory fitness and the reference-standardized clustered risk score. Mean (95% CI) of the reference-standardized clustered cardiometabolic risk score (excluding CRF) across quartiles of CRF. A higher clustered risk score indicates a less favorable cardiometabolic profile. Children in quartile 1 of CRF are the least fit and children in quartile 4 the fittest. *P* for trend < 0.001.

Cardiorespiratory fitness was significantly inversely associated with the reference-standardized clustered risk score (excluding CRF) (β - 0.37 SD, 95% CI –0.43 to –0.31), adjusted by sex and pubertal stage. There were no significant moderating effect of sex for the association between CRF and cardiometabolic clustered risk. Fig 2 illustrates the inverse association between quartiles of CRF (quartile 1 represents the least fit children and quartile 4 the most fit children) and the cardiometabolic reference-standardized clustered risk score in girls and boys (*p for trend* < 0.001).

## Discussion

This is the first study to produce standardized cardiometabolic risk values according to the international reference material for cardiometabolic risk factors in children published by Stavnsbo et al. [10]. This method makes it possible to compare otherwise sample-specific clustered risk scores directly to international age- and sex-specific cardiometabolic risk values. The Norwegian children in the present study had a higher clustered risk score of traditional risk factors than the international reference population, due to higher levels of SBP, HOMA score, and TG. However, CRF was considerably higher in the Norwegian children and including CRF in the clustered risk score decreased the score to below international values.

Children from Nordic countries have earlier exhibited higher SBP values compared to children from other European countries [28, 29], which the present study confirms. Elevated BP levels have been associated with organ damage in children [30] and track moderately into adulthood, increasing the risk of subclinical atherosclerosis [31]. Systolic BP was included in the clustered risk score, although DBP was lower in the Norwegian children compared to the international reference values. Systolic BP has been argued for as a recommended measure in children due to a greater accuracy and reproducibility than measures of DBP [32], and is often used as a single component to represent BP levels in pediatric clustered risk scores [10]. In addition, SBP has been shown to be a better predictor of cardiometabolic risk compared to DBP in adults [33]. Norwegian adults have also shown to have higher SBP than many other European countries and the US [34]. Dietary differences between countries could possibly cause these variations in BP [35]. However, methodological differences could also be a plausible explanation for these findings. For instance, approximately 50% of the total population included in the reference material was drawn from the National Health and Nutrition Examination Survey (NHANES) from the USA, for which BP was measured by the use of a mercury sphygmomanometer. The auscultation method using a mercury sphygmomanometer is the “gold standard” for BP measures [36]. In the present study we used an oscillometric BP device to measure BP. When validated against the mercury sphygmomanometer, oscillometric BP devices have been shown to significantly overestimate SBP in children (2.53 mmHg; 95% CI [0.57 to 4.50]) [37]. In comparison, the difference between SBP values in the Norwegian 10-year-olds and the international age-comparable children was 2.48 mmHg (95% CI [1.93–3.02]). The accuracy, however, of BP measures also depends on other factors such as the surrounding environment in which the measures are completed, the measurement procedures, and the test personnel [38].

The children in the present study had significantly higher HOMA scores than the international reference population. HOMA score has been shown to be a good surrogate measure for insulin resistance in youth, when validated against the euglycemic-hyperinsulinemic clamp method [39]. Also, HOMA score has been shown to be a better predictor for clustered cardiometabolic risk factors, than fasting glucose and insulin levels alone [40]. During the last decades, prevalence rates of insulin resistance and type 2 diabetes in children and adolescents have been on the rise globally [41]. Comparing HOMA levels in the ASK study to population-based samples of 9-10-year-old Norwegian children from 2005–2006 [42], shows that Norwegian children may following international trends. When glucose and insulin was standardized separately according to the international reference values, the ASK population had slightly lower glucose values, but considerably higher insulin levels than the reference population. A potential methodological challenge when comparing insulin levels between populations is the use of different kits for analysis, since the binding characteristics of the plates may result slightly different. Storage time of blood samples before analyzes also influence on insulin levels. As an example of this, the multicultural European Youth Heart Study (EYHS), included in the reference material, had their Portuguese blood samples stored for several years before analysis. The Danish samples were analyzed shortly after collection in a WHO-certified laboratory. A subgroup of the Danish samples were reanalyzed to investigate the influence of the long storage time, showing a strong correlation between insulin values between the first and second analysis (*r* = 0.97), but a substantial decrease (≈ 50%) in insulin levels. Thus, insulin levels from EYHS-Portugal were corrected for storage time to apply to all other obtained insulin values in the EYHS study. It is, nevertheless, plausible that differences in storage time of blood samples in the studies included in the reference material and in the ASK study, could contribute to the higher insulin values and HOMA score observed in the present study.

Pediatric dyslipidemia is associated with initiation and progression of atherosclerotic lesions [43] and has shown persistence over time [44], increasing the risk of early atherosclerosis and premature cardiovascular diseases. Triglycerides and TC:HDL-ratio were included in the clustered risk score in the present study to represent dyslipidemia, and both have been widely used in clustered risk scores in pediatric populations [10]. Compared to the international reference population, TG levels were significantly higher in the Norwegian children. In stratified analysis, girls exhibited higher TG levels than the Norwegian boys. One explanation of this finding could be that TG concentrations are positively associated with sexual maturation [45], and girls have been shown to enter puberty at younger ages than observed previously [46]. For instance, the Copenhagen Puberty Study found that thelarche among Danish girls in 2006 started nearly one year earlier than it had 15 years previously, independently of changes in BMI [47]. The Norwegian children did not differ from the international reference population in TC:HDL-ratio, despite significantly higher HDL-C levels. High levels of HDL-C are positively correlated with CRF, which was higher in the present study than the international reference values, and HDL-C has been shown to have an anti-atherosclerotic, and to some extent also cardioprotective, effect [48]. Lipid and lipoproteins levels are, nonetheless, influenced by several environmental and genetic factors, such as diet [49] and apolipoprotein variants [50], which we were not able to control for.

Time trends in cardiometabolic risk factor levels in children and the fact that the reference values are based on pooled data from both European and US data, may explain some of the observed differences between the Norwegian and international risk factor values in general. The reference material comprised studies conducted between 1999 and 2008, while baseline data from the ASK study was collected in 2014. For instance, obesity levels in children and adolescents have shown a significant linear increase among children and adolescents during the last decades in both Europe and the US [51, 52]. The prevalence of obesity in Europe has, nevertheless, not increased to the same extent as in the US. According to the WHO [51], the mean prevalence of obesity among adolescents (11-, 13- and 15-year-olds) from 27 European countries was 4% in 2014. In comparison, the prevalence of obesity among US children aged 12–15 years in 2014 was 20% [52]. Further, the WHO reported that the lowest level of obesity in Europe in 2014 was found among Norwegian adolescents and Norway was one of the only European countries where an overall decrease in obesity was observed in the years between 2002 and 2014 (although only significant in 13-year-old boys) [51]. This might explain why the children in the present study had significantly lower WCs than the international reference children. The Norwegian children’s BMI did, however, not differ from the reference population, but WC was prioritized in the clustered risk score since WC has been shown to be a stronger predictor of cardiometabolic risk than is BMI [53–55]. Waist circumference is strongly inversely associated with CRF [16] and CRF levels in the present study were significantly higher than in the reference population.

Among the examined variables, CRF (VO_2peak_) clearly differed the most from the international values, 1.20 and 1.23 SDs in girls and boys, respectively. Thus, when we included CRF (inversed) in the clustered risk score, the risk score decreased considerably. Cardiorespiratory fitness is associated with cardiometabolic health in both children [13, 14, 16, 17] and adults [56, 57]. In adults, low CRF has shown to be a stronger predictor of CVDs and all-cause mortality than other established risk factors, such as hypertension, type 2 diabetes and high cholesterol levels [11, 12, 56–58]. Furthermore, both epidemiological studies and clinical evidence show that CRF in addition to other traditional cardiometabolic risk factors enhances the precision of predicting CVD morbidity and mortality [56]. Although different fitness tests were used to produce reference values for CRF, these fitness tests rely on solid validations to reflect absolute VO_2peak_ [10]. In addition, VO_2peak_ in the present study was estimated from validated algorithms, but could possibly overestimate true VO_2peak_ values [24]. However, high CRF levels is in line with previous studies showing that Norwegian children have relatively high VO_2peak_ [42, 59]. In support of this findings, international comparisons of children´s PA levels show that Norwegian children are more physically active and spend more time in higher intensities than children from most other countries [60].

It is rather contradictory that the Norwegian children have higher clustered cardiometabolic risk levels despite lower WC and higher CRF, compared to international values. One would expect lower (healthier) cardiometabolic risk factor levels in a fit population. However, similar findings were reported in a previous study from Western Norway [16]. The reason for the discrepancy in these risk factor profiles is difficult to manifest, but it could be due to cultural or environmental factors or to any of the reasons discussed earlier, such as diet, genetics or methodologically differences. The ASK population is both lean and fit, and despite having higher levels of some cardiometabolic risk factors compared to international values, these levels are still considered to be within a healthy range. Importantly, cardiometabolic risk factor levels seems in general to have improved in children from Western Norway during the last decade [16].

The present study found an inverse association between CRF and clustered cardiometabolic risk (*r* = - 0.37) in accordance with earlier findings [13–17]. In comparison, Andersen et al. [13] found a stronger association (*r* = - 0.49) than was observed in the present study, while others have found weaker associations (*r* = - 0.31 to—0.09) [15, 17]. Aadland and colleagues [14] recently showed that CRF measured by the Andersen test is a more accurate marker of cardiometabolic health compared to directly measured VO_2peak_ and time to exhaustion (TTE), determined from a graded treadmill protocol in Norwegian 10-year-old children. The standardized regression coefficient between the Andersen test and clustered cardiometabolic risk in the present study was lower than found by Aadland et al. [14]; *r* = - 0.45. Still, the Andersen test in the present study performed slightly better than both VO_2peak_ and TTE presented by Aadland et al. [14] as a marker of cardiometabolic health. Cardiorespiratory fitness is not regarded as one of the traditional cardiometabolic risk components and has been proclaimed as an overlooked and underutilized risk factor for cardiometabolic diseases along with PA [56, 57]. The undisputable positive association of CRF with cardiometabolic health, however, strongly implicates its importance and argues for the inclusion of CRF in clustered cardiometabolic risk scores.

The main strength of the present study was the use of international reference values to standardize the cardiometabolic risk factors. This approach makes it possible to directly compare otherwise population specific clustered risk scores to the reference material of international cardiometabolic risk values. More specifically, if other studies adapt the same standardization strategy as the present study, they could compare single and clustered risk scores both to the international reference values and the present study population. Thus, the strength of using the reference values will increase with its usage in different studies and populations, and will make it possible to look at secular trends in the future. Further, the relatively large population sample is a strength of the present study. However, the homogeneity within the group (i.e. rural children from one Norwegian county, primarily Caucasian and in a limited age-range), limits generalization of the results. The use of the Andersen aerobic fitness test to estimate VO_2peak_ could be a limitation of our findings, since the Andersen test is an indirect measure of CRF. However, the test has shown both validity and reliability in the target age-group [24, 61]. As discussed earlier, the Andersen test also performed better as a marker of cardiometabolic health than both a direct VO_2peak_ test and TTE [14]. Furthermore, this test is more feasible in large population studies than direct measurement of VO_2peak_. The Andersen test is a suitable aerobic fitness test in children because the intermittent running reflects children’s natural running pattern and because it does not stigmatize children with a low CRF level. Dietary status was not registered in the ASK study and poses a limitation to the present study, since nutrition is a contributing factor to cardiometabolic risk [62].

## Conclusions

This study is the first to standardize cardiometabolic risk factor levels in children according to international reference values. In comparison with international values, Norwegian children had significantly more favorable WC, DBP, glucose, HDL-C and CRF levels, but similar or less favorable levels of other cardiometabolic risk factors. The clustered cardiometabolic risk score (excluding CRF) was higher in the Norwegian children compared to the reference population. However, the Norwegian children`s CRF levels were more than 1 SD higher than the international mean. Adding CRF to the clustered cardiometabolic risk score lowered the score to below international values. CRF was associated with cardiometabolic risk, with low fit children scoring significantly higher on clustered cardiometabolic risk factors than children with higher CRF levels.

## Supporting information

S1 Dataset Supplementary data file including all material underlying the present study. (SAV)
